# Compromises with Nature

**Published:** 1876-07

**Authors:** Ben. H. Pratt


### THE BISTOURY.

Vol. XII. ELMIRA, N. Y., JULY, 1876. No. 2.

### Contributions.

### COMPROMISES WITH NATURE.

BEN. H. PRATT.

Man was created a conditional being. His physical, mental and moral perfections were not assured except so far as he complied with certain general formulæ; and in proportion to his observance or neglect of these, he will prove a physical, mental and moral giant or pigmy. The germs of faculty are innate in every normal human being anterior to birth. As within the seed which we plant in the earth exist the germs of root, stem, leaf, blossom and fruit; and as the development of these depend upon conditions of moisture, heat, sunlight and cultivation; so within each child born into the world are germs of perfect manhood or womanhood.

To develop this idea, a volume would not suffice, hence, in a brief paper like this, we may only glance at a single point in the vast field of living and being, exhibiting, if we may be able, the fact that man does not kindly submit to the conditions of healthful existence, and is therefore an ailer, or a deformity, or a monstrosity. In the matter of his physical development—and we will limit our reflections at this time to this element of his being—man seeks to compromise with his Creator. The condition upon which mankind is vouchsafed health and strength of body is the free and continued exercise of every muscle of his anatomy. To this end he was placed upon the earth with scarce any natural want made attainable except through the medium of a vigorous exercise of his bodily powers. He craves, first, sustenance; but he scarcely finds a single edible at hand that does not require labor to make it available for food. Even under the most favorable circumstances, the fruits hang beyond his reach—the succulent roots are below the earth's surface—the flesh of animals is invested with that which must be overcome. A sense of shame suggests clothing; but what to him are the ripened flax and cotton fields, or the fleece upon the backs of living creatures, or the silkworm's store of shining fibre? Nothing is his that he does not take and develop, even in the most fruitful climate and under the most favorable conditions that man can be surrounded by. Everything about him suggests labor, exercise, work. Nature gave him the germs of muscular health and strength, and says more plainly than lips can utter it—labor and live—do naught and die. On every hand, this conditional creature man finds provision made for the exercise of his physical being, and no man can say—no man dare say, without scandalizing his God, "there is no work for me to do." By reason of the spirit of antagonism in the human mind, however, there come, in progress of time, and as the result of an almost divinely endowed prescience, expedients by which labor, as a necessity—that is, to supply the actual wants of man—can be dispensed with, and the wise of the earth are able to use the foolish as their proxies. The necessity for labor being wanting, ease suggests idleness, and idleness superinduces a dwarfing and enervation by which the physical being lapses into disease, and as its result we have all those ills to which the human race is subject. Hence is demonstrated the fact, that whether we will or no, to have health, even in a moderate degree, we must exercise our bodies. The law is imperative, and either death or life-long misery must result from a disobedience of the law.

But, by some peculiar quirk of society, that which nature has pronounced essential—that which human wisdom accepts as necessary to health—that which God himself has proclaimed the condition of life—this has become ignoble and debasing. How to avoid work and yet enjoy a modicum of health, has become the great problem of modern times. Human ingenuity is constantly on the rack to devise some means of thwarting the spirit of the divine law and yet obeying the letter thereof—some means of getting around this very troublesome fiat of Deity—some means of somehow fooling nature, and making her believe we are obeying her laws, when we are actually plotting the overthrow of her government, and endeavoring to usurp her place by a creature of our own devising. We have a hatred of Work—we are enamored of Play. We desire to supplant the former by the latter, and make life one round of play for those whose necessities do not demand work. The carrying out of this grand scheme is what is now agitating one-half of civilized mankind, and it may not be amiss to note some of the expedients that are resorted to, in order to effect a consummation so grandly beautiful in theory, but so inadequate in practice.

To secure the physical benefits which all admit are the natural result of work, and yet to do no work—to convert into a pleasure, or an excitement, that exercise which nature decreed shall be expended in work—to work, and yet to deceive those who despise work into believing that we are only playing—we resort to play. Tired of rest—ennuied by reason of the monotony of listlessness—growing weaker and weaker day by day, and conscious of the inroads of creeping disease, owing to the lack of that muscular exertion upon which the integrity of our physical being depends, we hire a man to nail a board upon our garden fence, while we swing Kehoe clubs for exercise; employ a man to spade our garden beds, while we resort to health-lifts for exercise; pay a sawyer to prepare our kindling wood, while we resort to an hour's rowing for lung expansion; send for a man to take up and shake and put down our carpets, while we endanger our lives and limbs in a game of base-ball; keep a hired man to groom our horse, while we repair to the gymnasium for a season of dangerous play; send our children to calisthenic schools, and forbid their doing chores about the house; sneer at the suggestion of papering or painting a room in our houses, and get into a profuse perspiration over the swinging of a pair of dumb-bells; ridicule the idea of carrying a parcel four blocks, and walk a mile every fifteen minutes with a billiard cue in our hands; take a street car to get a quarter of a mile from home, and walk twenty miles a day, over rocks and fallen trees, and through swamps and ditches and bramble-fields to catch fifty cents worth of trout with a fifty-dollar rod and line; feel awfully imposed upon if requested to bring home two pounds of steak for supper, but will carry a fifteen-pound gun, with pounds more of powder and shot, and submit to be loaded down with accouterments during a whole day's tramp through the woods for a pair of quail; dance five hours at an evening party, to get to which, though it be but fifty steps distant, one must needs call a carriage; hire a small boy to black our boots, and then go to a ten-pin alley to labor and sweat, and get mad over the knocking down of a few bottle-shaped pins by means of ponderous balls; work one's self into a fever hammering wooden balls with a croquet mallet, and disdaining the idea of wielding a broom-handle for half an hour; lace, and tug, and pull, and pin, and tie, and crimp, and frizzle, and comb, and powder, and paint, and pinch one's toes, and deform one's self, and get into a fume and fret over the preparations for an evening ball, but turn up the nose in contemptuous sneer if required to sew up two yards of carpet-seam; fuss and stew and blow at a picnic fire to get the coffee hot, until one's face is as red as a boiled lobster, and then stir the freezer until one's arm don't get the tremble out of it for twenty four hours, but grow white with indignant rage at being asked to broil the family breakfast steak, or peep into the oven to see how the biscuit browns; in fine do anything, however laborious, however irksome, however disagreeable, however nasty, however disreputable the same thing would be if it were really work, and do it with the blandest possible manner, provided it is not work—not a necessary act—not essential to one's sustenance—does not result in any practical accomplishment.

The great tendency of the age seems to be an effort to abolish work and resort to play, to secure so much of the wealth of the world as will gratify all necessary wants, in order that one may play during the balance of his natural life. The grand secret of happiness is thus overlooked, while the means of sustaining health are not secured. Work is made essential to health, not only because it affords the necessary muscular exercise, but because the result of achieved work, taken in connection with the labor given, brings a mental satisfaction that seems to complement the physical exertion, and the two combined result healthfully; for physical development, without a corresponding mental strength, is not healthful, any more than excessive mental cultivation, to the neglect of the physical, is healthful. The difference between work and play is nowhere except in results. The former shows something accomplished that is plainly useful—of advantage to somebody—and in contemplation of the end sought and the good it will confer upon the world, or our community, or family, or even upon a single individual, lie fully one-half the excellences of the labor expended. Play is well nigh purposeless, and there is no pleasure in the contemplation of results, because these are only temporary and evanescent.

Play, however, is not to be decried, for as the alternation of work its benefits are beyond computation. All work and no play is as bad as all play and no work, and he or she is happiest who can so alternate labor and recreation as to make each the more intensely enjoyable by reason of its proper connection with the other. They who are so fortunate as to have found their work not irksome but a joy in itself, and to which they can go with a species of longing—work that gives pleasure in itself, and not because of the emolument it affords—work that the worker would rather do than take part in the most attractive play he is acquainted with—work that no prospect of ease can lure from, and which rises to the full height and spreads out to the full breadth of the worker's ambition—these have found their pearl of great price and are the only really enviable mortals here below. Such a coincidence of work and inclination is the mother of contentment, and contentment, be it in the most rural hamlet or the folk-surcharged metropolis, is the only happiness known to humanity. Why do people covet ease when ease is full of unknown ills? Why will the comfortably endowed envy the wealthy, when wealth has a thousand cares and annoyances that competence does not dream of? Simply beause they mistakenly construe work into a curse, and ease into the acme of all human bliss—a sentiment that is as wide of the truth as the zenith is wide of the antipodes.

